# Magnetic resonance imaging before breast cancer surgery: results of an observational multicenter international prospective analysis (MIPA)

**DOI:** 10.1007/s00330-021-08240-x

**Published:** 2021-10-13

**Authors:** Francesco Sardanelli, Rubina M. Trimboli, Nehmat Houssami, Fiona J. Gilbert, Thomas H. Helbich, Marina Álvarez Benito, Corinne Balleyguier, Massimo Bazzocchi, Peter Bult, Massimo Calabrese, Julia Camps Herrero, Francesco Cartia, Enrico Cassano, Paola Clauser, Andrea Cozzi, Danúbia A. de Andrade, Marcos F. de Lima Docema, Catherine Depretto, Valeria Dominelli, Gábor Forrai, Rossano Girometti, Steven E. Harms, Sarah Hilborne, Raffaele Ienzi, Marc B. I. Lobbes, Claudio Losio, Ritse M. Mann, Stefania Montemezzi, Inge-Marie Obdeijn, Umit A. Ozcan, Federica Pediconi, Katja Pinker, Heike Preibsch, José L. Raya Povedano, Daniela Sacchetto, Gianfranco P. Scaperrotta, Simone Schiaffino, Margrethe Schlooz, Botond K. Szabó, Donna B. Taylor, Özden S. Ulus, Mireille Van Goethem, Jeroen Veltman, Stefanie Weigel, Evelyn Wenkel, Chiara Zuiani, Giovanni Di Leo

**Affiliations:** 1 Department of Biomedical Sciences for Health, Università degli Studi di Milano, Milan, Italy; 2 Unit of Radiology, IRCCS Policlinico San Donato, Via Rodolfo Morandi 30, 20097 San Donato Milanese, Italy; 3 Sydney School of Public Health, Faculty of Medicine and Health, The University of Sydney, Sydney, Australia; 4 Department of Radiology, School of Clinical Medicine, Cambridge Biomedical Campus, University of Cambridge, Cambridge, UK; 5 Department of Biomedical Imaging and Image-guided Therapy, Division of General and Pediatric Radiology, Research Group: Molecular and Gender Imaging, Medical University of Vienna, Vienna, Austria; 6 Department of Radiology, Hospital Universitario Reina Sofía, Córdoba, Spain; 7 Department of Radiology, Institut Gustave Roussy, Villejuif, France; 8 Institute of Radiology, Department of Medicine, Università degli Studi di Udine, Udine, Italy; 9 Department of Pathology, Radboud University Medical Center, Nijmegen, The Netherlands; 10 Unit of Breast Radiology, IRCCS Ospedale Policlinico San Martino, Genoa, Italy; 11 Department of Radiology, Hospital Universitario de La Ribera, Alzira, Spain; 12 Unit of Breast Imaging, Fondazione IRCCS Istituto Nazionale dei Tumori, Milan, Italy; 13 Breast Imaging Division, IEO, European Institute of Oncology IRCCS, Milan, Italy; 14 Department of Breast Surgery, Hospital Sírio Libanês, São Paulo, Brazil; 15 Department of Radiology, Hospital Sírio Libanês, São Paulo, Brazil; 16 Department of Radiology, MHEK Teaching Hospital, Semmelweis University, Budapest, Hungary; 17 Breast Center of Northwest Arkansas, Fayetteville, AR USA; 18 Department of Radiology, Di.Bi.MED, Università degli Studi di Palermo, Policlinico Universitario Paolo Giaccone, Palermo, Italy; 19 Department of Radiology and Nuclear Medicine, Maastricht University Medical Center, Maastricht, The Netherlands; 20 Department of Breast Radiology, IRCCS Ospedale San Raffaele, Milan, Italy; 21 Department of Radiology and Nuclear Medicine, Radboud University Medical Center, Nijmegen, The Netherlands; 22 Department of Radiology, The Netherlands Cancer Institute, Amsterdam, The Netherlands; 23 Department of Radiology, Azienda Ospedaliera Universitaria Integrata Verona, Verona, Italy; 24 Department of Radiology and Nuclear Medicine, Erasmus University Medical Center, Rotterdam, The Netherlands; 25 Unit of Radiology, Acıbadem Mehmet Ali Aydınlar University School of Medicine, İstanbul, Turkey; 26 Department of Radiological, Oncological and Pathological Sciences, Università degli Studi di Roma “La Sapienza”, Rome, Italy; 27 Department of Radiology, Breast Imaging Service, Memorial Sloan Kettering Cancer Center, New York, NY USA; 28 Department of Diagnostic and Interventional Radiology, University Hospital of Tübingen, Tübingen, Germany; 29 Kiwifarm S.R.L, La Morra, Italy; 30 Disaster Medicine Service 118, ASL CN1, Saluzzo, Italy; 31 CRIMEDIM, Research Center in Emergency and Disaster Medicine, Università degli Studi del Piemonte Orientale “Amedeo Avogadro”, Novara, Italy; 32 Department of Surgery, Radboud University Medical Center, Nijmegen, The Netherlands; 33 Department of Radiology, Barking Havering and Redbridge University Hospitals NHS Trust, London, UK; 34 Medical School, Faculty of Health and Medical Sciences, The University of Western Australia, Perth, Australia; 35 Department of Radiology, Royal Perth Hospital, Perth, Australia; 36 Gynecological Oncology Unit, Department of Obstetrics and Gynecology, Department of Radiology, Multidisciplinary Breast Clinic, Antwerp University Hospital, University of Antwerp, Antwerpen, Belgium; 37 Maatschap Radiologie Oost-Nederland, Oldenzaal, The Netherlands; 38 Institute of Clinical Radiology and Reference Center for Mammography, University of Münster, Münster, Germany; 39 Department of Radiology, University Hospital of Erlangen, Erlangen, Germany

**Keywords:** Breast cancer, Magnetic resonance imaging, Mastectomy, Breast-conserving surgery, Reoperation

## Abstract

**Objectives:**

Preoperative breast magnetic resonance imaging (MRI) can inform surgical planning but might cause overtreatment by increasing the mastectomy rate. The Multicenter International Prospective Analysis (MIPA) study investigated this controversial issue.

**Methods:**

This observational study enrolled women aged 18–80 years with biopsy-proven breast cancer, who underwent MRI in addition to conventional imaging (mammography and/or breast ultrasonography) or conventional imaging alone before surgery as routine practice at 27 centers. Exclusion criteria included planned neoadjuvant therapy, pregnancy, personal history of any cancer, and distant metastases.

**Results:**

Of 5896 analyzed patients, 2763 (46.9%) had conventional imaging only (noMRI group), and 3133 (53.1%) underwent MRI that was performed for diagnosis, screening, or unknown purposes in 692/3133 women (22.1%), with preoperative intent in 2441/3133 women (77.9%, MRI group). Patients in the MRI group were younger, had denser breasts, more cancers ≥ 20 mm, and a higher rate of invasive lobular histology than patients who underwent conventional imaging alone (*p* < 0.001 for all comparisons). Mastectomy was planned based on conventional imaging in 22.4% (MRI group) versus 14.4% (noMRI group) (*p* < 0.001). The additional planned mastectomy rate in the MRI group was 11.3%. The overall performed first- plus second-line mastectomy rate was 36.3% (MRI group) versus 18.0% (noMRI group) (*p* < 0.001). In women receiving conserving surgery, MRI group had a significantly lower reoperation rate (8.5% versus 11.7%, *p* < 0.001).

**Conclusions:**

Clinicians requested breast MRI for women with a higher a priori probability of receiving mastectomy. MRI was associated with 11.3% more mastectomies, and with 3.2% fewer reoperations in the breast conservation subgroup.

**Key Points:**

*• In 19% of patients of the MIPA study, breast MRI was performed for screening or diagnostic purposes.*

*• The current patient selection to preoperative breast MRI implies an 11% increase in mastectomies, counterbalanced by a 3% reduction of the reoperation rate.*

*• Data from the MIPA study can support discussion in tumor boards when preoperative MRI is under consideration and should be shared with patients to achieve informed decision-making.*

## Introduction

In patients newly diagnosed with breast cancer, the routine use of breast magnetic resonance imaging (MRI) before surgery is a controversial topic [1, 2], attracting extensive debate and little consensus [3–5]. Proponents reasonably draw on the established evidence of MRI sensitivity to detect additional disease, allowing more tailored surgical planning [6, 7]. Opponents point out the lack of evidence on clinical benefit from preoperative MRI and raise concerns that it causes more mastectomies than needed [8–10]. Guidelines are heterogenous, ranging from defined but limited indications [11, 12] to recommendations against [13].

The Multicenter International Prospective Analysis (MIPA) study was undertaken to provide new knowledge on this topic, building evidence on whether and to what extent MRI impacts surgical treatment in breast cancer practice.

## Methods

### Study design and population

The MIPA study was registered in the International Standard Randomized Controlled Trial Number Register (ISRCTN41143178). Methods, detailed in the protocol paper [14], are summarized here.

The study was initiated by the European Network for the Assessment of Imaging in Medicine, endorsed by the European Society of Breast Imaging, and conducted in accordance with the Declaration of Helsinki. It was approved on January 29, 2013, by the Ethics Committee of the coordinating center (protocol number 2784) and thereafter by local Ethics Committees of participating centers. All participants signed an informed consent, unless waived by local Ethics Committees.

The study was coordinated and monitored by the principal investigator at IRCCS Policlinico San Donato, San Donato Milanese, Italy. The principal investigator (first author) and the lead statistician (last author) had full access to the database, generated statistical analyses, prepared the first manuscript draft, and assume responsibility for the accuracy and completeness of the data and for the adherence of the study to the protocol.

MIPA is a large-scale observational study enrolling women with needle biopsy-proven breast cancer. Enrolled patients underwent or did not undergo MRI before surgery as part of routine practice at each center, resulting in two concurrent groups ex post: women who underwent digital mammography/tomosynthesis and/or breast ultrasonography, i.e., conventional imaging (noMRI group), and women who received MRI in addition to conventional imaging (MRI group). Following a public call, centers were selected for participation among those that documented high breast MRI volumes and the use of protocols recommended by international societies [11, 12, 15]. All MRI examinations were performed before/after administration of macrocyclic or linear contrast agents (in Europe, only before the linear ones were banned). Gadobutrol (Bayer AG) at a dose of 0.1–0.2 mmol/kg of body weight was used in 22/27 centers.

The study population included women aged 18–80 years newly diagnosed with breast cancer and amenable to upfront surgery. Exclusion criteria were an indication to neoadjuvant therapy, personal history of breast or any other cancer, distant metastases at diagnosis, pregnancy, and inability to provide consent.

MRI examinations were classified as performed with preoperative (ipsilateral local staging and contralateral screening), diagnostic (problem-solving), or bilateral screening purposes.

### Endpoints

Primary endpoints were the first-line mastectomy rate (endpoint 1) and the immediate/short term reoperation rate for close or positive margins (endpoint 2), calculated amongst all patients. Secondary surgical endpoints were the first-line only bilateral mastectomy rate (endpoint 3), and the overall mastectomy rate (endpoint 4), obtained summing first-line mastectomies and conserving surgeries converted into mastectomies. For the MRI group, other secondary surgical endpoints were the additional mastectomy rate and the rate of change to more or less extensive conserving surgery (wider excision or multiple excisions). Secondary clinical endpoints (rate of breast recurrence and distant metastases) will be evaluated at a 5-year follow-up.

### Sample size and statistical analysis

The sample size calculation, set at 7000 patients, has been described in the protocol paper [14].

Statistical analysis followed a per-protocol approach. Only patients having an electronic case report form with all data needed for the endpoints’ calculation were analyzed. Statistical analysis was performed on a per-patient basis whenever possible. In this regard, patients receiving mastectomy on one side and conserving surgery on the other side, as well as those receiving bilateral mastectomy, were considered as mastectomy patients.

The MRI and noMRI groups were compared in terms of baseline characteristics including demographics, high familial breast cancer risk (three or more first-degree relatives with breast or ovarian cancer), breast density, tumor size, histopathology at needle biopsy, and surgical plan based on conventional imaging only, as defined by the multidisciplinary team. To allow a reasonable comparison between the two groups, patients receiving MRI for screening or diagnostic purposes were excluded from comparative analysis, since surgical planning based on conventional imaging was not possible.

Given that MIPA is a nonrandomized study, the inferential analysis used statistical tests to ascertain whether the two groups were homogenous. Depending on distributions, comparisons of continuous variables were performed using the Student *t* test or the Mann–Whitney *U* test for independent data, or using the *χ*^2^ test for categorical variables.

Baseline characteristics were included as covariates in statistical modelling. A binary logistic regression analysis was performed using Nagelkerke *R*^2^ as a measure of the endpoint variability that was explained by the analyzed predictors. Predictors were added one by one and kept in the final model only if increasing *R*^2^ or if the associated *p* value was < 0.05. Odds ratios (ORs) for such predictors with their 95% CIs were calculated. The final model’s area under the curve was calculated using receiver operating characteristic analysis.

In the MRI group, the mastectomy rate planned before and after MRI was calculated: the difference was the measure of the additional mastectomy rate associated with MRI.

## Results

**Fig. 1:**
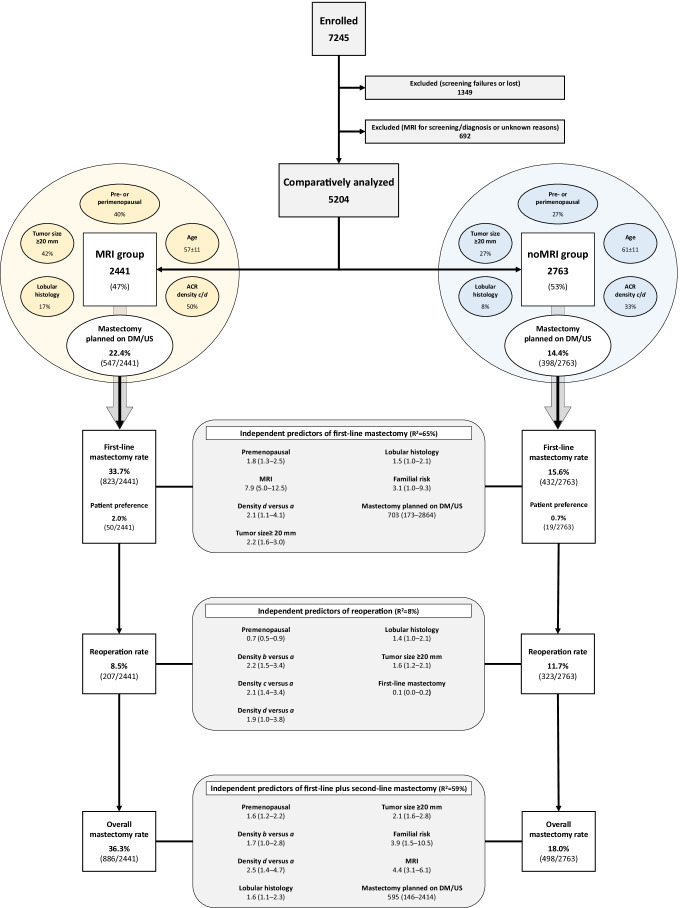
Study flowchart and results regarding predictors of surgical outcome. For each predictor, odds ratio and 95% confidence interval are reported. *R*^2^ represents the Nagelkerke goodness of fit. MRI, magnetic resonance imaging; DM, digital mammography; US, ultrasonography

Patient enrollment began in June 2013 and ended in November 2018, achieving a total of 7245 patients in 27 participating centers worldwide, enrollment per center being detailed in the protocol paper [14]. Of the 7245 enrolled patients, 1349 (18.6%) were excluded a priori from analysis, being screening failures (i.e., one or more exclusion criteria discovered only after enrollment), treated with neoadjuvant therapy after enrollment, patients who moved to another center, or patients who had missing data. Among the remaining 5896 patients, 3133/5896 (53.1%) underwent MRI in addition to conventional imaging, with the following purposes: diagnostic in 496/3133 women (15.8%), bilateral screening in 111/3133 women (3.5%), unknown in 85/3133 women (2.8%), and explicitly preoperative in 2441/3133 women (77.9%), this last group being the MRI group considered for comparison. On the other hand, 2763/5896 women (46.9%) received conventional imaging only (noMRI group). Preoperative MRI was ordered by: radiologists alone in 1309/2441 women (53.6%); surgeons alone in 808/2441 women (33.1%); radiologists and surgeons in 216/2441 women (8.8%); radiologists, surgeons, and oncologists in 65/2441 women (2.7%); other combinations of physicians in 43/2441 women (1.8%). The enrolment flowchart is shown in Fig. 1.

### Between-group comparison of baseline characteristics

Table 1 reports differences in characteristics between groups. The MRI group included younger women (57 ± 11 versus 61 ± 11 years, *p* < 0.001) and a larger proportion of pre- or perimenopausal women (40.0% versus 26.6%, *p* < 0.001), women with dense breasts (50.1% versus 33.2%, *p* < 0.001), invasive lobular histology at percutaneous biopsy (17.4% versus 7.9%, *p* < 0.001), and cancers ≥ 20 mm on final pathology (42.4% versus 27.5%, *p* < 0.001).

**Table 1:** Patient and tumor characteristics in the MIPA study

Patient or tumor characteristic	MRI group	noMRI group	*p*
Age at diagnosis—years^a^	57 ± 11	61 ± 11	< 0.001
Pre- or perimenopausal (%)	972/2432 (40.0%)	731/2748 (26.6%)	< 0.001
Dense breast: ACR density category *c* or *d* (%)	1171/2336 (50.1%)	831/2503 (33.2%)	< 0.001
Three or more first-degree relatives with history of breast or ovarian cancer (%)	35/2432 (1.4%)	34/2749 (1.2%)	0.526
BRCA1/2 mutation	20/2431 (0.8%)	14/2748 (0.5%)	0.161
Multifocal/multicentric breast cancer on DM^b^ (%)	307/2143 (14.3%)	212/2435 (8.7%)	< 0.001
Diameter of the largest lesion on DM and/or US—mm^c^	16 (11 to 24)	15 (10 to 21)	< 0.001
Ductal carcinoma in situ at biopsy (%)	409/2421 (16.9%)	472/2554 (18.5%)	0.255
Invasive lobular histology at biopsy (%)	341/1959 (17.4%)	164/2078 (7.9%)	< 0.001
Triple-negative breast cancer (%)	103/2234 (4.6%)	107/2391 (4.5%)	0.585
Tumor size on final pathology ≥ 20 mm (%)	799/1886 (42.4%)	537/1953 (27.5%)	< 0.001

Distributions are given per patient, unless differently specified. Variations of denominators are due to different rates of unknown data

*ACR* American College of Radiology; *DM* digital mammography; *US* ultrasound

^a^ Mean ± standard deviation

^b^ Per-breast distribution

^c^ Per-breast distribution, given as median and interquartile interval

### Planned mastectomy

Mastectomy planned on the basis of conventional imaging only was more frequent in the MRI group (22.4% versus 14.4%), with a crude OR of 1.7 (95% CI, 1.5 to 2.0). Similarly, bilateral mastectomy was planned on the basis of conventional imaging in 31/516 women (6.0%) of the MRI group and in 8/398 women (2.0%) of the noMRI group, with a crude OR of 2.9 (95% CI, 1.3 to 6.4).

**Fig. 2:**
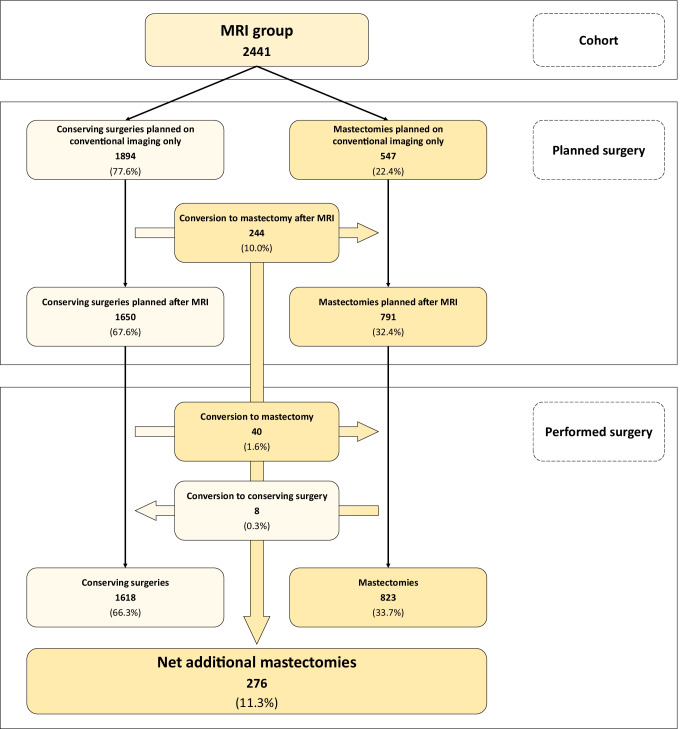
Surgical planning and performed surgery in the MRI group. MRI, magnetic resonance imaging

**Fig. 3:**
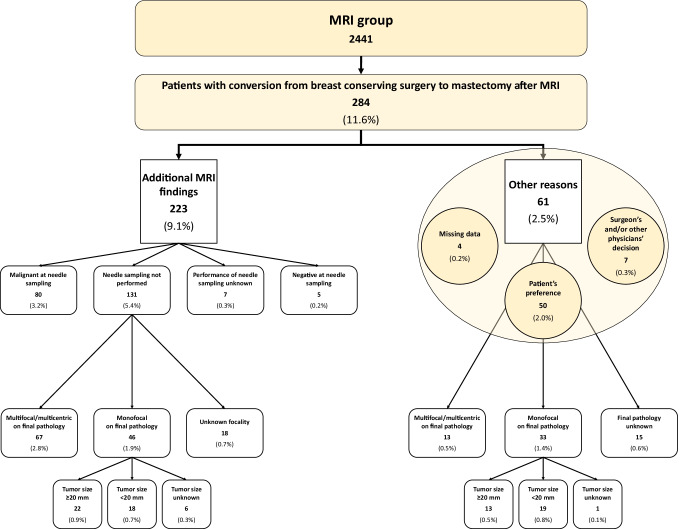
Reasons for conversion from breast-conserving surgery to mastectomy in the MRI group. MRI, magnetic resonance imaging

In the MRI group, mastectomy was already planned on conventional imaging in 547/2441 women (22.4%), in 791 women after MRI (32.4%), and was ultimately performed in 823/2441 women (33.7%) (Fig. 2). These 276 additional mastectomies (from 547 to 823) in the MRI group included patient’s preference in 50/2441 women (2.0%) and recommendations from surgeons and/or other physicians in 7/2441 women (0.3%) (Fig. 3). Moreover, this 11.3% additional mastectomy absolute rate associated with MRI (from 22.4 to 33.7%) was the difference between an 11.6% rate of conversion from conserving surgery to mastectomy (284/2441 women) and a 0.3% rate of opposite conversion, from mastectomy to conserving surgery (8/2441 women). The 284/2441 (11.6%) conversions from conserving surgery to mastectomy were prompted in 223/2441 cases by additional MRI findings (9.1%) and by other reasons in 61/2441 cases (2.5%), mainly dominated by patient’s preference (50/2441 cases, 2.0%). As shown in Fig. 3, these conversions were supported by malignancy at needle sampling in 80/223 cases (35.9%), with 67/131 (51.1%) cases in which needle sampling was not performed being found to be multifocal or multicentric on final pathology.

Considering the 1641 breasts that received conserving surgery in the MRI group (with available data for this analysis), the surgical extent was as planned on conventional imaging in 1387/1641 cases (84.5%). The remaining 254 women received less extensive surgery (26/1641, 1.6%), more extensive single excision (186/1641, 11.3%), or multiple excisions (42/1641, 2.6%).

### First-line unilateral and bilateral mastectomy

In the noMRI group, the rate of mastectomies that were actually performed as first-line surgery (endpoint 1) increased from the 14.4% planned on conventional imaging to the 15.6% that was actually performed. The MRI group had a more than double mastectomy rate compared to the noMRI group (33.7% versus 15.6%), with a crude OR of 2.7 (95% CI, 2.4 to 3.1). These percentages include the patient’s preference to receive mastectomy: 2.0% in the MRI group and 0.7% in the noMRI group. Logistic regression analysis (Fig. 1 and Table 2) showed that MRI was an independent risk factor for mastectomy (OR 7.9), together with the highest breast density (American College of Radiology [ACR] category *d* versus category *a*, OR 2.1), invasive lobular histology at biopsy (OR 1.5), high familial risk (OR 3.1), premenopausal status (OR 1.8), lesion diameter ≥ 20 mm (OR 2.2), and planned mastectomy on conventional imaging (OR 703). The area under the curve of this model was 0.914.

**Table 2:** Logistic regression model of variables associated with first-line mastectomy

Variable	T	Standard error	Wald	*p*	Odds ratio (95% CI)
MRI	2.071	0.230	80.780	< 0.001	7.93 (5.05–12.46)
High familial risk^a^	1.133	0.560	4.097	0.043	3.10 (1.04–9.29)
Premenopausal	0.560	0.172	10.550	0.001	1.75 (1.25–2.46)
ACR breast density category *b*	0.199	0.275	0.524	0.469	1.22 (0.71–2.09)
ACR breast density category *c*	0.151	0.287	0.277	0.599	1.16 (0.66–2.04)
ACR breast density category *d*	0.762	0.331	5.302	0.021	2.14 (1.12–4.10)
Lobular histology	0.372	0.198	3.519	0.061	1.45 (0.98–2.14)
Tumor size on final pathology ≥ 20 mm	0.781	0.156	25.174	< 0.001	2.18 (1.61–2.96)
Planned mastectomy on conventional imaging	6.556	0.716	83.784	< 0.001	703.47 (172.81–2863.61)
Constant	-4.685	0.308	231.567	< 0.001	0.009

Nagelkerke *R*^2^ = 65.3%. Area under the curve = 0.914

*ACR* American College of Radiology; *CI* confidence interval; *MRI* magnetic resonance imaging

^a^ Three or more first-degree relatives with breast or ovarian cancer

The rate of first-line bilateral mastectomy among women receiving mastectomy (endpoint 3) was 87/823 (10.6%) in the MRI group (30/823 cases, 3.6%, due to patient’s preference) and 12/432 (2.8%) in the noMRI group (2/432 cases, 0.5%, due to patient’s preference), with a crude OR of 4.1 (95% CI, 2.2 to 7.7). In the logistic regression analysis (Table 3), MRI (OR 3.6) and high familial risk (OR 4.7) were the only independent risk factors for bilateral mastectomy, while mastectomy planned on conventional imaging was a protective factor (OR 0.6).

**Table 3:** Variables associated with first-line bilateral mastectomy in the subgroup of breast cancer patients receiving first-line mastectomy

Variable	T	Standard error	Wald	*p*	Odds ratio (95% CI)
MRI	1.291	0.323	15.960	< 0.001	3.64 (1.93–6.85)
High familial risk^a^	1.554	0.471	10.905	0.001	4.73 (1.88–11.90)
Planned mastectomy on conventional imaging	–0.508	0.223	5.193	0.023	0.60 (0.38–0.93)
Constant	–3.165	0.354	80.058	< 0.001	0.042

Nagelkerke *R*^2^ = 7.9%. Area under the curve = 0.669

*CI* confidence interval; *MRI* magnetic resonance imaging

^a^ Three or more first-degree relatives with breast or ovarian cancer

### Reoperation and overall mastectomy rate

The reoperation rate for close/positive margins (endpoint 2) was lower in the MRI group than in the noMRI group (8.5% versus 11.7%, *p* < 0.001), with a crude OR of 0.70 (95% CI, 0.58 to 0.84). In the logistic regression analysis (Fig. 1 and Table 4), breast density was a risk factor for reoperation: compared to ACR density *a*, OR was 2.2 for *b*, 2.1 for *c*, and 1.9 for *d*. Invasive lobular histology (OR 1.4) as well as lesion diameter ≥ 20 mm were also independent risk factors (OR 1.6). Vice versa, premenopausal status (OR 0.7) and received mastectomy as first-line surgery (OR 0.1) were protective factors against reoperation.

**Table 4:** Logistic regression model of variables associated with reoperation

Variable	T	Standard error	Wald	*p*	Odds ratio (95% CI)
Premenopausal	–0.428	0.165	6.709	0.010	0.65 (0.47–0.90)
ACR breast density category *b*	0.788	0.216	13.244	< 0.001	2.20 (1.44–3.36)
ACR breast density category *c*	0.758	0.232	10.646	0.001	2.14 (1.35–3.37)
ACR breast density category *d*	0.636	0.351	3.289	0.070	1.89 (0.95–3.76)
Lobular histology	0.360	0.183	3.868	0.049	1.43 (1.00–2.05)
Tumor size on final pathology ≥ 20 mm	0.460	0.137	11.314	0.001	1.58 (1.21–2.07)
First-line mastectomy	–2.337	0.392	35.493	< 0.001	0.10 (0.05–0.21)
Constant	–2.798	0.202	191.550	< 0.001	0.061

Nagelkerke *R*^2^ = 7.7%. Area under the curve = 0.672

*ACR* American College of Radiology; *CI* confidence interval

The overall mastectomy rate (endpoint 4) was higher in the MRI group than in the noMRI group (36.3% versus 18.0%), with a crude OR of 2.6 (95% CI, 2.3 to 2.9). As shown in Fig. 1 and Table 5, premenopausal status (OR 1.6), breast density (OR 1.7 for ACR density *b* and 2.5 for *d*, compared to ACR density *a*) high familial risk (OR 3.9), invasive lobular histology (OR 1.6), MRI (OR 4.4), lesion diameter ≥ 20 mm (OR 2.1), and planned mastectomy on conventional imaging (OR 595) increased the odds of mastectomy.

**Table 5:** Logistic regression model of variables associated with overall mastectomy

Variable	T	Standard error	Wald	*p*	Odds ratio (95% CI)
MRI	1.472	0.170	75.022	< 0.001	4.36 (3.12–6.08)
High familial risk^a^	1.372	0.498	7.599	0.006	3.95 (1.49–10.47)
Premenopausal	0.478	0.155	9.571	0.002	1.61 (1.19–2.18)
ACR breast density category *b*	0.539	0.253	4.529	0.033	1.72 (1.04–2.82)
ACR breast density category *c*	0.423	0.266	2.523	0.112	1.53 (0.91–2.57)
ACR breast density category *d*	0.927	0.313	8.761	0.003	2.53 (1.37–4.67)
Lobular histology	0.467	0.178	6.859	0.009	1.60 (1.13–2.26)
Tumor size on final pathology ≥ 20 mm	0.738	0.140	27.926	< 0.001	2.09 (1.59–2.75)
Planned mastectomy on conventional imaging	6.386	0.716	79.616	< 0.001	594.65 (146.00–2414.15)
Constant	–4.128	0.261	249.319	< 0.001	0.016

Nagelkerke *R*^*2*^ = 59.4%. Area under the curve = 0.884

*ACR* American College of Radiology; *CI* confidence interval; *MRI* magnetic resonance imaging

^a^ Three or more first-degree relatives with breast or ovarian cancer

## Discussion

This study reports actual clinical practice with breast MRI before breast cancer surgery. Of 5896 patients, about half received MRI. However, MRI was performed for screening, diagnosis, or unknown purpose in 692/5896 patients (over one in five patients), i.e., not originally intended as “preoperative.” In the 2441/5896 patients having MRI performed with preoperative intent (MRI group, 41.4%), MRI was requested by radiologists alone (53.6%) or surgeons alone (33.1%), reflecting current clinical practice [16, 17]. MRI was preferentially ordered for younger and premenopausal patients, for patients with dense breast tissue, with lobular cancers, or cancers ≥ 20 mm, as a survey already suggested [17].

Our data outline the contribution of conventional imaging to mastectomy indication in patients who also had MRI and define factors predicting surgical outcomes, including but not limited to MRI. Considering the overall (first- *plus* second-line) mastectomy rate, the higher rate (36.3%) found in the MRI group compared to the noMRI group (18.0%) represents a major finding for surgical oncology. The role of premenopausal status, breast density, familial risk, cancer diameter ≥ 20 mm, and invasive lobular histology in increasing the mastectomy risk (see Table 1) was expected [18, 19]. Importantly, a planned mastectomy on conventional imaging inherently made mastectomy almost unavoidable, with all but eight conventional imaging-based mastectomies confirmed after MRI. Conventional imaging had already suggested mastectomy in 66.5% of the women who ultimately received such surgery, showing that MRI was often used as a confirmation tool. Indeed, women with a mastectomy planned on conventional imaging had a 1.7-fold probability to receive MRI compared to those with a conserving surgery planned on conventional imaging, while the additional first-line mastectomy rate after MRI was curtailed to 11.3%.

In addition, the higher first-line bilateral mastectomy rate in the MRI group (10.6% versus 2.8%), decreasing to 7.0% versus 2.3% when subtracting bilateral surgeries due to patients’ preference, was partially driven by patient selection. Indeed, logistic regression analysis confirmed the role of both familial risk and MRI in predicting first-line bilateral mastectomy, with an OR of 4.7 and 3.6, respectively. Moreover, mastectomy planned on conventional imaging acted as a protective factor against first-line bilateral mastectomy. These findings derive from the high MRI sensitivity also for contralateral lesions and from a selection to MRI of patients with a higher propensity to bilateral mastectomy. Indeed, women with a planned bilateral mastectomy on conventional imaging had a 2.9-fold probability of receiving MRI compared to those with unilateral mastectomy planned on conventional imaging.

Regarding conversions from conserving surgery to mastectomy attributed to additional MRI findings, we note that only 80/223 (35.9%) cases were confirmed as malignant by needle sampling, with other 5/223 (2.2%) cases being reported as negatives. For the remaining 138/223 cases (61.9%), we must consider the crucial point of an accurate three-dimensional radiological-pathological correlation between MRI findings and overall mastectomy specimens, a task very difficult to be performed in real-world clinical practice [20].

In our study, the MRI group had an absolute 3.2% lower reoperation rate compared to the noMRI group (8.5% and 11.7%, respectively), as already hinted by two randomized controlled trials [21, 22]. The POMB trial [21], investigating a relatively young population, reported a significant reduction in reoperation rate from 15 to 5%, while the IRCIS trial [22], assessing ductal carcinoma in situ, reported a non-significant reduction from 27 to 20%. Breast density, invasive lobular histology, and diameter ≥ 20 mm negatively impacted our overall reoperation rate, as already reported by other authors [23–25]. Vice versa, premenopausal status and, again as expected, first-line mastectomy reduced the odds for reoperation. However, MRI did not act as an independent reducer of reoperation risk.

Hence, the MIPA study brings new insights in comparison with previous randomized studies [26, 27] and meta-analyses [4] which did not report any reduction in reoperation rate in their MRI groups. However, the reduction in the reoperation rate demonstrated in our MRI group must be read in the light of the increase in mastectomy rate, as shown by the protective role of the first-line mastectomy against reoperation. This reasoning also applies to our finding that mastectomy planned on conventional imaging protected against reoperation. The trade-off between first-line mastectomy and reoperation is a matter for debate with relevant issues in terms of psychological impact, complication rates, and final cosmetic results [28, 29]. Regarding conserving surgery, MRI did not alter the surgical planning based on conventional imaging in 84.5% of cases. MRI-based conserving surgery was more extensive than planned on conventional imaging in 13.9% and less extensive in 1.6% of cases. The question of whether MRI-tailored surgery will benefit patients at follow-up awaits future evaluation of our MIPA cohorts, including the assessment of breast recurrence and distant metastases rates.

The strengths of this observational study are related to its real-world multicenter large-scale size [30], which allowed us to take into consideration real-world data on 5896 patients. The real-world data approach of the MIPA study reflects current clinical practice, providing insights on a variety of issues, frequently overlooked in the discussion about preoperative MRI, such as the selection of patients referred for preoperative MRI and the difficulties in obtaining a lesion-by-lesion radiological-pathological correlation in everyday practice.

The first limitation of this study, i.e., its non-randomized design, is indeed counteracted by the approach of the MIPA study with a large and diverse study population. Other limitations of the study are represented by the exclusion of 1349 patients from analysis and the selection of centers with considerable clinical experience and high breast MRI volumes, potentially limiting the generalizability of our results.

Notwithstanding the aforementioned limitations, the results of our study—which provides quantitative information about the probability of conversion from breast-conserving surgery to mastectomy and the reduction of the reoperation rate—may be usefully taken into account in tumor board meetings and may contribute to enhance the awareness and involvement of breast cancer patients when MRI is being considered before surgery.

In conclusion, the MIPA study evaluated the clinical practice of performing or not performing breast MRI in a large cohort of breast cancer patients. Across 27 centers worldwide, 53% of patients underwent MRI, with a 4.4 OR of receiving mastectomy when compared to patients who underwent conventional imaging only, counterbalanced by a 3% lower reoperation rate. Mastectomy was already planned on the basis of conventional imaging in 14% of the noMRI group and 22% of the MRI group, where MRI was frequently used as a confirmation tool toward mastectomy, leading to an 11% increase in mastectomy rate, 9% when excluding patient’s preference.

## Abbreviations

ACR: American College of Radiology
CI: Confidence interval
MRI: Magnetic resonance imaging
OR: Odds ratio

## Declarations

### Guarantor

The scientific guarantor of this publication is Prof. Francesco Sardanelli, MD.

### Statistics and biometry

The third author (Nehmat Houssami, The University of Sydney) and the last author (Giovanni Di Leo, IRCCS Policlinico San Donato) have significant statistical expertise.

### Methodology

- Prospective
- Observational
- Multicentre study
